# Fibrinogen-like Protein 1 as a Predictive Marker for the Incidence of Severe Acute Pancreatitis and Infectious Pancreatic Necrosis

**DOI:** 10.3390/medicina58121753

**Published:** 2022-11-29

**Authors:** Yuhang Sui, Zhongjie Zhao, Yang Zhang, Tao Zhang, Guanqun Li, Liwei Liu, Hongtao Tan, Bei Sun, Le Li

**Affiliations:** 1 Department of Pancreatic and Biliary Surgery, The First Affiliated Hospital of Harbin Medical University, Harbin 150001, China; 2 Department of Abdominal Surgery, Guiqian International General Hospital, Guiyang 550024, China; 3 Key Laboratory of Hepatosplenic Surgery, Ministry of Education, Harbin 150001, China

**Keywords:** acute pancreatitis, fibrinogen-like protein 1, infected pancreatic necrosis, prediction, diagnosis

## Abstract

*Background and Objectives*: Acute pancreatitis (AP) is defined as an acute inflammatory disorder of the pancreas and is a common gastrointestinal disease. Since currently used indicators lack specifics and cannot accurately reflect the phase of disease, better diagnostic approaches need to be explored. Fibrinogen-like protein 1 (FGL-1) is a reactant in acute inflammatory diseases and is increased in the plasma of AP patients. In the current study, we aim to investigate the clinical benefits of FGL-1 in predicting the severity of AP and infected pancreatic necrosis (IPN), which can improve the diagnostic efficiency of AP. *Materials and Methods*: In this study, 63 patients diagnosed with AP from December 2018 to September 2019 were enrolled. Regarding the severity of AP, patients were separated into severe acute pancreatitis (SAP, *n* = 12) and No-SAP groups (*n* = 51). On the basis of infective conditions, patients were divided into IPN (*n* = 9) and No-IPN (*n* = 54) groups. The demographic data (sex and age) and blood parameters (WBC, HCT, glucose, calcium, FIB, APTT, PCT, CRP, and FGL-1) were retrospectively analyzed. *Results*: The plasma FGL-1 levels were increased in both SAP (*p* < 0.01) and IPN (*p* < 0.05) subgroups compared to the healthy control group. Multivariate analysis showed that elevated plasma FGL-1 (*p* < 0.01) and PCT levels (*p* < 0.05) within 72 h after the onset of AP were positively correlated with the severity of AP, while increased plasma FGL-1 (*p* < 0.01) and CRP (*p* < 0.05) levels were positively correlated with the occurrence of IPN. The combination of FGL-1 and PCT showed superiority to both individual markers in SAP prediction. However, the combination of FGL-1 and CRP showed no diagnostic advantage over CRP in IPN prediction. *Conclusions*: Plasma FGL-1 within 72 h after the onset could be used for the stratification of AP and its infectious complications. The combination of PCT and FGL-1 presents an enormous advantage for the early identification of SAP.

## 1. Introduction

Acute pancreatitis (AP) is an inflammatory disease caused by activated trypsin, leading to pancreatic autodigestion, edema, and even infection or necrosis. AP is a common acute abdomen disease in emergency rooms that can be stratified to mild, moderate, and severe according to the revised Atlanta Classification [1]. Mild pancreatitis is usually presented as a self-limiting disease from which patients can recover within one week. The prolonged and recurrent AP can develop to chronic pancreatitis or pancreatic intraepithelial neoplasia, ultimately increasing the risk of pancreatic cancer [2]. Severe acute pancreatitis (SAP) is characterized by rapid onset and higher mortality ranging from 15% to 30% [3,4,5]. The occurrence of multiple organ failure (MOF) and infected pancreatic necrosis (IPN) are major risk factors for poor prognosis. Due to lacking predictability of diagnosis and management, the best timing of therapy may be delayed. Thus, it is urgent to discover biomarkers for early and timely diagnosis that may effectively predict the severity and complications of AP.

The severity score classification systems which are commonly used in AP include Ranson, Glasgow, Acute Physiology and Chronic Health Evaluation II (APACHE-II), and CT severity index (CTSI) (or modified CTSI). However, there are limited uses in clinics due to complicated operation and lack of timeliness [6]. In recent years, the early achievable severity index (EASY) has been considered as a practical tool for identifying patients with a high risk of SAP, with an average area under curve (AUC) score of 0.81 ± 0.033 and accuracy of 89.1%. Nevertheless, none of the single parameters, as well as complex evaluation of multiple clinical, radiological, and biochemical parameters, can precisely identify patients with a high risk of SAP in the early phase of disease. Many studies have explored useful biomarkers for evaluating the severity of AP and predicting related complications. Several biomarkers have been selected as candidates for predicting patients’ outcome, such as interleukin-6 (IL-6), C-reactive protein (CRP), procalcitonin (PCT), and copeptin [7,8,9,10]. CRP and PCT are regarded as the most promising parameters, but their sensitivity and specificity are still limited [7,11]. Fibrinogen-like protein 1 (FGL-1), a member of the fibrinogen family, is derived from hepatocytes, and plays crucial roles in hepatic steatosis and insulin resistance [12]. Similar to CRP, FGL-1 is an acute-phase reactant synthesized by hepatocytes that responses to local and systemic inflammatory processes. Moreover, IL-6 increases FGL-1 expression and directly exacerbates downstream acute inflammatory response [13]. A previous study has shown increased circulating FGL-1 levels in acute alcoholic pancreatitis, but few studies have discussed the association between elevated FGL-1 level and the stratification of inflammatory diseases [14].

In the current study, the plasma level of FGL-1 was tested in AP patients within the onset time point of 72 h. Herein, we investigate the efficacy of FGL-1 to evaluate the severity of AP and the occurrence of IPN.

## 2. Materials and Methods

### 2.1. Patients

As a retrospective study, a total of 67 patients diagnosed with AP in the First Affiliated Hospital of Harbin Medical University from December 2018 to September 2019 were recruited; 63 (39 males and 24 females) patients fulfilled the inclusion criteria and were finally enrolled, and 4 patients were excluded due to incomplete clinical data. All patients met the criteria for AP according to the revised Atlanta 2012 criteria. SAP is defined as persistent organ failure lasting for more than 48 h with or without local or systemic complications. The diagnostic criteria for organ failure was following the modified Marshall score system (with a score of 2 or more indicating the presence of organ failure in any system). IPN diagnosis can be suspected through the patient’s clinical course (fever) or the presence of gas within the collection in CECT. Patients with pancreatic parenchymal necrosis and/or peripancreatic necrosis, including acute necrotic collection (ANC) in the early stage and walled-off necrosis (WON) in the late stage, were diagnosed with IPN. Fine-needle aspiration is a valuable procedure for the evaluation of necrosis. The inclusion criteria of AP patients were as follows: (1) diagnosed with AP; (2) older than 18 years old; (3) first onset and within 72 h. The exclusion criteria were as follows: (1) pregnant women and children; (2) patients with malignancies; (3) combined with immune deficiency disease or autoimmune pancreatitis; (4) patients with incomplete clinical data. Patients were divided into SAP and No-SAP groups by incidence of organ failure and its duration. Regarding the basis of clinical symptoms, imaging, and pus puncture bacterial culture, patients were divided into IPN and No-IPN groups. The clinical data of the patients’ age, sex, and other general data are shown in Table 1. All participated patients signed informed consent, and this study was approved by the ethics committee of the First Affiliated Hospital of Harbin Medical University.

### 2.2. Clinical Data Collection

Clinical data of patients who met the inclusion criteria were collected: (1) general data, including sex, age, smoking history, onset time of abdominal pain, length of hospital, etiology and complications (heart disease, diabetes and hypertension); (2) laboratory parameters, such as white blood cells (WBC), hematocrit (HCT), activated partial thromboplastin time (APTT), fibrinogen (FIB), blood glucose, blood calcium, CRP and PCT in plasma for the first time after admission; (3) FGL-1 determination: the plasma of AP patients within the time point of onset for 72 h was collected and spun at 1000× *g* 4 °C for 15 min, then stored at −80 °C for use. The plasma FGL-1 concentration was determined by Human Fibrinogen-Like Protein 1 (FGL1) ELISA Kit (Cusabio, Wuhan, Hunan, China). Briefly, FGL1 antibody was precoated in a microplate. Standards and samples (50 μL) and detection antibody solution were loaded. The plate was incubated for 2 h at room temperature. After removing unbound substances, the avidin-conjugated horseradish peroxidase (HRP) was added and incubated for 30 min. Then, the stop solution was added and the optical density (OD) was obtained from a spectrophotometer using 450 nm as reference and 630 nm as correction wavelength (Thermo Fisher Scientific, Waltham, MA, USA). The standard curve was generated and the concentration of samples were calculated.

### 2.3. Statistical Analysis

SPSS software (IBM SPSS25.0, SPSS Inc., Chicago, IL, USA) was used for data analysis, and ROC curves were plotted using GraphPad Prism 9.0. The categorical variables were expressed in numbers and percentages (n, %), while Pearson chi-square test or Fisher exact probability test were used for significance analysis. Continuous variables were expressed as mean and standard deviation (x ± SD) or median and interquartile range (IQR). A non-parametric Mann–Whitney test of skewness distribution variables or a *t*-test of normal distribution variables was used for different intergroup comparison. Independent predictors were determined by a multiple logistic regression model. *p* < 0.05 was recognized as statistically significant.

## 3. Results

### 3.1. Patient Characteristics

A total of 63 patients diagnosed with AP were enrolled in this study. According to 2012 Atlanta Classification, patients were divided into MAP (*n* = 48), MSAP (*n* = 3) and SAP (*n* = 12). Since only three patients comprised the diagnosed-with-MSAP group, they were not defined as a subgroup. Patients were divided into a SAP group (*n* = 12) and No-SAP group (*n* = 51). Subsequently, patients were divided into an IPN group (*n* = 9) and No-IPN group (*n* = 54) according to whether they were complicated with IPN.

**Figure 1:**
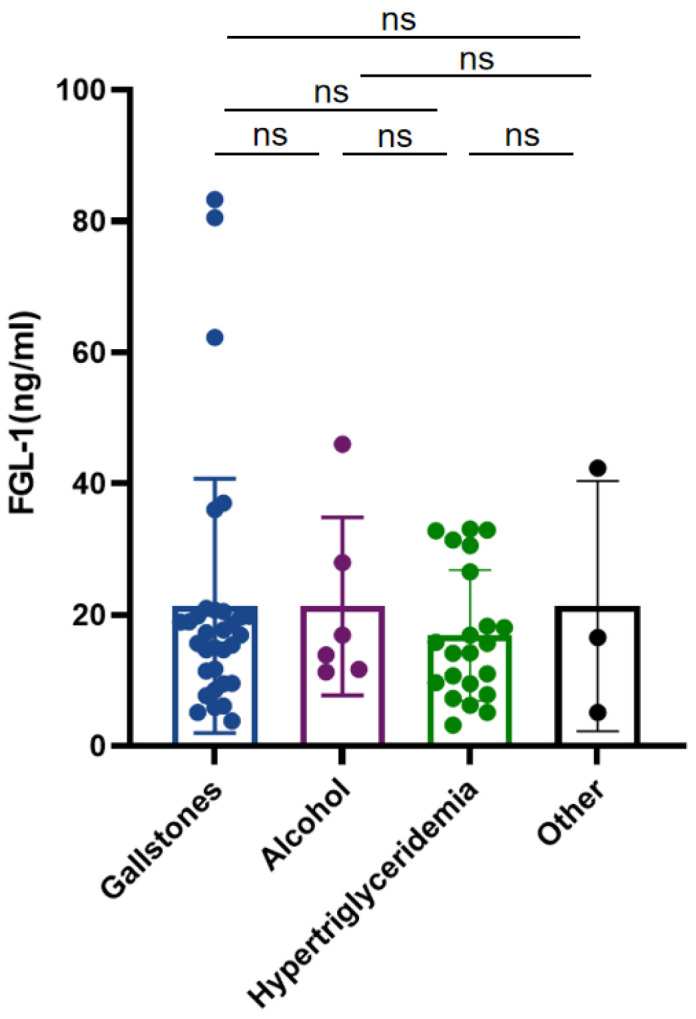
Comparison of Fibrinogen-like protein 1 (FGL-1) levels among different etiologies in AP. ns: presents no significant difference.

No difference was found in gender, age, smoking history, complications and etiology between the SAP and No-SAP groups. The hospitalization time was longer in the SAP group compared to the No-SAP group (26.0 (10.5~34.8) vs. 8.0 (6.0~12.0), *p* < 0.001) (Table 1). No difference was found between the IPN and Non-IPN groups in gender, age, smoking history, complications and etiology. Similarly, the hospitalization time was longer in the IPN group than the No-IPN group (18.5 (33.0~50.0) vs. 8.0 (5.75~11.25), *p* < 0.001) (Table 2). Among all of the etiologies, gallstones were the main cause of AP, followed by hypertriglyceridemia. The FGL-1 level showed no difference among different etiologies (Figure 1).

### 3.2. FGL-1 and PCT Shows Excellent Diagnostic Power in SAP

**Figure 2:**
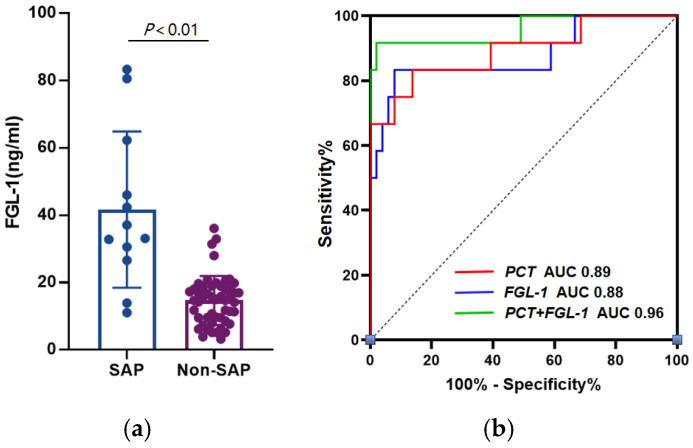
(**a**) Comparison of plasma Fibrinogen-like protein 1 (FGL-1) levels between severe acute pancreatitis (SAP) and Non-SAP groups; (**b**) Receiver operating characteristic (ROC) curves of FGL-1, procalcitonin (PCT) and combination model in the prediction of SAP.

It was found that the plasma FGL-1 level was significantly higher in the SAP group than the No-SAP group ((41.63 ± 23.20) ng/mL vs. (14.66 ± 7.27) ng/mL, *p* = 0.002) (Figure 2a). Univariate analysis revealed that plasma PCT (*p* = 0.002), CRP (*p* = 0.031) and FGL-1 (*p* < 0.001) levels within 72 h after the onset of AP showed a positive correlation with the onset of SAP (Table 3). Multivariate analysis indicated that PCT > 0.5 ng/mL (*p* = 0.049) and FGL-1 > 23.78 ng/mL (*p* = 0.001) were independent risk factors for SAP (Table 4). The ROC curves were then used to explore the efficacy of the above markers for predicting SAP (Figure 2b). The areas under the ROC curves of PCT and FGL-1 were 0.89 (95% CI, 0.77–1.00) and 0.88 (95% CI, 0.75–1.00), respectively. The sensitivity, specificity and cutoff value of PCT were 83.33%, 88.24% and 1.26 ng/mL, and those of FGL-1 were 83.33%, 94.12% and 23.78 ng/mL.

### 3.3. FGL-1 and CRP Shows Better Diagnostic Power in IPN

**Figure 3:**
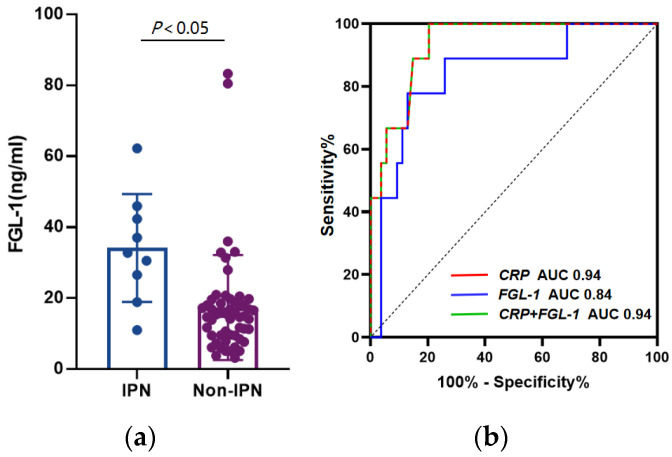
(**a**) Comparison of plasma Fibrinogen-like protein 1 (FGL-1) levels among different groups in severe acute pancreatitis (SAP); (**b**) Receiver operating characteristic (ROC) curves for FGL-1, C-reactive protein (CRP) and combination model in the prediction of infected pancreatic necrosis (IPN).

Our data revealed that the plasma FGL-1 level was significantly higher in the IPN group compared with the No-IPN group ((34.17 ± 15.19) ng/mL vs. (17.40 ± 14.80) ng/mL, *p* = 0.011) (Figure 3a). Univariate analysis demonstrated that plasma PCT, CRP and FGL-1 levels were positively correlated with the occurrence of IPN (*p* < 0.001) (Table 3). Multivariate analysis suggested that CRP > 430 mg/dL (*p* = 0.021) and FGL-1 > 23.79 ng/mL (*p* = 0.006) were independent risk factors for IPN (Table 4). Since both FGL-1 and CRP levels were increased in IPN patients, ROC curves were plotted to investigate the efficacy of either, or in combination, in IPN prediction (Figure 3b). The areas under the ROC curves of CRP and FGL-1 were 0.94 (95% CI, 0.87–1.00) and 0.84 (95% CI, 0.70–0.99), respectively, and the sensitivity, specificity and cutoff value of CRP were 100%, 79.63% and 397 mg/dL, and these of FGL-1 were 77.78%, 87.04% and 23.79 ng/mL.

### 3.4. Combination of FGL-1 and PCT Improves the Predictive Capability for SAP

In the prediction model of SAP, the areas under the ROC curves of PCT combined with FGL-1 was 0.96 (95% CI, 0.88–1.00), and the sensitivity and specificity were 91.67% and 98.04% (Figure 2b). In the prediction model of IPN, the areas under the ROC curves of CRP combined with FGL-1 was 0.94 (95% CI, 0.87–1.00), and the sensitivity and specificity were 100% and 79.63%, which was similar to CRP (Figure 3b). Furthermore, a combined diagnostic model was developed using logistic regression analysis. The AUC, sensitivity, specificity, accuracy, positive likelihood ratio (LR+), negative likelihood ratio (LR−), positive predictive value (PPV) and negative predictive value (NPV) of combined detection are shown in Table 5 and Table 6.

## 4. Discussion

AP is a potentially life-threatening inflammatory disease with two death peaks. Multiple organ failure caused by inflammatory reactions induces the first death peak. IPN-related infectious complications are the factors leading to sepsis or organ failure, which cause the second death peak in later stage [15,16,17]. The outcome of AP is rapidly changing and fluctuates unpredictably [18]. Currently, clinical scoring systems, for instance, Ranson, Glasgow and APACHE-II, are complicated to calculate and take at least 48 h to determine. These shortcomings reflect the urgency to figure out simple biochemical parameters to estimate the development of AP at an early stage, and effective intervention can be achieved prior to rapid exacerbation [19]. In addition, identifying mild diseases will avoid over-treatment and cut the economic burden.

Fibrinogen-like protein 1 (FGL-1), also known as hepassocin, is a member of the fibrinogen family. FGL-1 induces hepatocyte proliferation and repairs liver injury by activating the epidermal growth factor receptor (EGFR) and SRC-dependent pathways [20,21,22]. FGL-1 was initially found to be highly expressed in human liver. It also exists in plasma, and approximately 20% of FGL-1 is in an unbound free state, indicating that its biological effects may be systemic [12,23,24]. FGL-1 is increased in cross types of cancers, including lung cancer, pancreatic cancer, colorectal cancer and melanoma, and a higher level in tumors indicates poor prognosis. Additionally, FGL-1 is the main immune ligand of lymphocyte-activation gene 3 (LAG-3) that negatively regulates T cells’ activation and plays a vital role in hepatocyte regeneration, glucose and lipid metabolism, as well as serving as a potential target for cancer immunotherapy [25,26,27]. Moreover, FGL-1 is considered as a promoter of inflammatory processes and causes liver injury through activating the IL-6/STAT3 signaling pathway [12]. Protein omics suggest that FGL1 acts as a biomarker for predicting rheumatoid arthritis progression and could be used for evaluating the pathogenesis of Crohn’s disease [28,29]. These findings hint at the potential effects of FGL-1 in immune activation and inflammatory development in AP.

Our study revealed that the plasma FGL-1 level was significantly increased in SAP patients and those accompanied with IPN (*p* < 0.05) (Figure 2a and Figure 3a), and increased FGL-1 level was positively associated with SAP and IPN. FGL-1 level has been found to be elevated in acute alcoholic pancreatitis [14,23]. However, our data indicated no difference between different AP subtypes, suggesting that AP induced FGL1 elevation in an etiology-independent manner (Figure 1). Cholelithiasis is the top cause of AP in China, followed by alcoholic factors. Due to the changes in dietary pattern and lifestyle shift, the incidence of hypertriglyceridemic pancreatitis is increasing and becoming the second leading cause [30]. PCT and CRP are satisfactory biomarkers and indicate beneficial effects of predicting the severity of AP and incidence of IPN. CRP, with a concentration over 150 mg/dL, is regarded as an accurate index to evaluate the incidence of SAP, and the sensitivity and specificity are 80~86% and 61~84% [7,8]. PCT has been considered as an early indicator of systemic bacterial infection, sepsis and organ failure [9]. The sensitivity and specificity for SAP prediction are 73% and 87% when the concentration is over 0.5 ng/mL [11]. In the current study, CRP showed no benefit in SAP prediction, while a higher PCT level presented positive relevance to SAP (*p* < 0.05) (Table 4). The possible causes could be either the plasma FGL-1 level reaching the peak around 72 h or that it is susceptible to other inflammatory diseases. Rau et al. have found that PCT > 3.5 ng/mL and CRP > 430 mg/dL are indicators for IPN. The sensitivity and specificity of PCT are 93% and 88% and those of CRP are 40% and 100%, which delineate the superiority of PCT over CRP [31]. Nevertheless, PCT showed no more power than CRP in our study (Table 6).

Our findings indicated that FGL-1 and PCT had exactly the same sensitivity (83.33%) in SAP prediction, and the specificity of FGL-1 was higher than PCT (94.12% vs. 88.24%). The combination was superior to both single indexes (AUC: 0.96, sensitivity: 91.67%, specificity: 98.04%). In the occurrence of IPN, the sensitivity of CRP was more remarkable than FGL-1 (100% vs. 77.78%), but the specificity was relatively poor (79.63% vs. 87.04%). In addition, the efficacy of the combination was consistent with CRP alone, demonstrating less necessity to integrate both markers for IPN evaluation. FGL-1 acts as a key regulator of organ failure in SAP patients. A previous study has reported that the activation of fibrin-like protein 2 (FGL-2), as the homologous protein of FGL-1, leads to the deposition of fibrin and the formation of microthrombosis, which exhibits the severity of AP [32]. Since FGL-1 and FGL-2 share parts of similar biological properties, further studies need to be raised to elucidate their biological roles in AP progression.

This study’s limitations are that it was a single-center study with a small sample size; a multi-center and large-sample study needs to be launched. Nevertheless, the current study presents novel non-invasive markers and views of tracking the development of AP, which will improve AP patients’ outcome.

## 5. Conclusions

In summary, our study discovers that increased plasma level of FGL-1 within 72 h after the onset is associated with the severity of AP. The combination of FGL-1 and PCT is superior to single indexes in SAP prediction, which provides a new approach for the stratification of AP.

## Figures and Tables

**Table 1:** Demographic data and clinical characteristics of SAP and No-SAP groups.

	SAP (*n* = 12)	No SAP (*n* = 51)	*p*-Value
Age—years	50.5 (39.8–62.0)	49.0 (41.0–64.0)	0.951
Gender—M/F	8/4	31/20	0.706
Comorbidities—no. (%)			
Heart disease	1/12 (8.33%)	9/51 (17.65%)	0.427
Diabetes	2/12 (16.67%)	14/51 (27.45%)	0.440
Hypertension	4/12 (33.33%)	13/51 (25.49%)	0.582
hLOS—days	26.0 (10.5–34.8)	8.0 (6.0–12.0)	<0.001
Etiology—no. (%)			
Gallstones	4/12 (33.33%)	28/51 (54.90%)	0.327
Alcohol	2/12 (16.67%)	4/51 (7.84%)	0.349
Hypertriglyceridemia	5/12 (41.67%)	17/51 (33.33%)	0.586
Other	1/12 (8.33%)	2/51 (3.92%)	0.518
Smoking history—no. (%)	4/12 (33.33%)	12/39 (30.77%)	0.483

Abbreviations: SAP, severe acute pancreatitis; hLOS, length of hospital stay.

**Table 2:** Demographic data and clinical characteristics of IPN and No-IPN groups.

	IPN (*n* = 9)	No IPN (*n* = 54)	*p*-Value
Age—years	44.0 (39.0–56.0)	49.0 (41.0–64.0)	0.298
Gender—M/F	7/2	32/22	0.290
Comorbidities—no. (%)			
Heart disease	0/9 (0%)	9/54 (16.67%)	0.225
Diabetes	3/9 (33.33%)	13/54 (24.07%)	0.555
Hypertension	2/9 (22.22%)	15/54 (27.78%)	0.728
hLOS—days	18.5 (33.0–50.0)	8.0 (5.75–11.25)	<0.001
Etiology—no. (%)			
Gallstones	3/9 (33.33%)	29/54 (53.70%)	0.258
Alcohol	1/9 (11.11%)	5/54 (9.26%)	0.861
Hypertriglyceridemia	4/9 (44.44%)	18/54 (33.33%)	0.517
Other	1/9 (11.11%)	2/54 (3.70%)	0.334
Smoking history—no. (%)	2/9 (22.22%)	14/54 (25.93%)	0.910

Abbreviations: IPN, infected pancreatic necrosis; hLOS, length of hospital stay.

**Table 3:** Univariate and multivariate analyses for identifying potential predictors of SAP.

		Severity		Univariate Analysis		Multivariate Analysis		
Variable	*n* = 63	SAP (*n* = 12)	No-SAP (*n* = 51)	*X* ^2^	*p*-Value	OR	95% CI	*p*-Value
WBC (10^9^/mL)				0.459	0.549	-	-	-
≤10	27	4	23					
>10	36	8	28					
HCT (%)				0.165	0.685	-	-	-
≤45	45	8	37					
>45	18	4	14					
Blood glucose (mmol/L)				0.932	0.334	-	-	-
≤7	19	5	14					
>7	44	7	37					
Ca^2+^ (mmol/L)				1.281	0.285	-	-	-
<2.25	42	10	32					
≥2.25	21	2	19					
FIB (g/L)				0.466	0.496	-	-	-
≤4.66	37	6	31					
>4.66	26	6	20					
APTT (s)				0.046	0.830	-	-	-
≤27.2	28	5	23					
>27.2	35	7	28					
PCT (ng/mL)				9.908	0.002	0.047	0.002~0.991	0.049
≤0.5	31	1	30					
>0.5	32	11	21					
CRP (mg/dL)				4.632	0.031	2.241	0.165~30.384	0.544
≤150	28	2	26					
>150	35	10	25					
FGL-1 (ng/mL)				32.029	<0.001	0.013	0.001~0.157	0.001
≤23.78	49	2	47					
>23.78	14	10	4					

Abbreviations: SAP, severe acute pancreatitis; WBC, white blood cell; HCT, hematocrit; FIB, fibrinogen; APTT, activated partial thromboplastin time; PCT, procalcitonin; CRP, C-reactive protein; FGL-1, Fibrinogen-like protein 1.

**Table 4:** Univariate and multivariate analyses for identifying potential predictors of IPN.

		IPN		Univariate Analysis		Multivariate Analysis		
Variable	*n* = 63	IPN (*n* = 9)	No-IPN (*n* = 54)	*X* ^2^	*p*-Value	OR	95% CI	*p*-Value
WBC (10^9^/mL)				0.389	0.533	-	-	-
≤10	27	3	24					
>10	36	6	30					
HCT (%)				0.117	0.733	-	-	-
≤45	45	6	39					
>45	18	3	15					
Blood glucose (mmol/L)				0.050	0.823	-	-	-
≤7	19	3	16					
>7	44	6	38					
Ca^2+^ (mmol/L)				2.333	0.127	-	-	-
<2.25	42	8	34					
≥2.25	21	1	20					
FIB (g/L)				0.884	0.347	-	-	-
≤4.66	37	4	33					
>4.66	26	5	21					
APTT (s)				0.525	0.469	-	-	-
≤27.2	28	3	25					
>27.2	35	6	29					
PCT (ng/mL)				14.860	<0.001	0.447	0.038~5.264	0.522
≤3.5	57	5	52					
>3.5	6	4	2					
CRP (mg/dL)				13.585	<0.001	0.060	0.006~0.649	0.021
≤430	50	3	47					
>430	13	6	7					
FGL-1 (ng/mL)				20.935	<0.001	0.034	0.003~0.384	0.006
≤23.78	50	2	48					
>23.78	13	7	6					

Abbreviations: IPN, infected pancreatic necrosis; WBC, white blood cell; HCT, hematocrit; FIB, fibrinogen; APTT, activated partial thromboplastin time; PCT, procalcitonin; CRP, C-reactive protein; FGL-1, Fibrinogen-like protein 1.

**Table 5:** Diagnostic value of FGL-1 and PCT and methodological comparison in SAP.

	AUC (95% CI)	Cut-Off	Sensitivity	Specificity	PPV	NPV	LR+	LR−
PCT (ng/mL)	0.89 (0.77–1.00)	1.26	83.33%	88.24%	62.50%	95.74%	7.09	0.19
FGL-1 (ng/mL)	0.88 (0.75–1.00)	23.78	83.33%	94.12%	76.92%	96.00%	19.23	0.24
FGL-1 + PCT	0.96 (0.88–1.00)	-	91.67%	98.04%	91.67%	98.04%	46.77	0.08

Abbreviations: AUC, area under curve; CI, confidence interval; PPV, positive predictive value; NPV, negative predictive value; LR+, positive likelihood ratios; LR−, negative likelihood ratios; PCT, procalcitonin; FGL-1, Fibrinogen-like protein 1.

**Table 6:** Diagnostic value of FGL-1 and CRP and methodological comparison in IPN.

	AUC (95% CI)	Cut-Off	Sensitivity	Specificity	PPV	NPV	LR+	LR−
CRP (mg/dL)	0.94 (0.87–1.00)	397	100%	79.63%	45.00%	100%	4.91	0
FGL-1 (ng/mL)	0.84 (0.70–0.99)	23.79	77.78%	87.04%	50.00%	95.92%	6.00	0.26
FGL-1 + CRP	0.94 (0.87–1.00)	-	100%	79.63%	91.67%	98.04%	4.91	0

Abbreviations: AUC, area under curve; CI, confidence interval; PPV, positive predictive value; NPV, negative predictive value; LR+, positive likelihood ratios; LR−, negative likelihood ratios; CRP, C-reactive protein; FGL-1, Fibrinogen-like protein 1.

## Data Availability

Not applicable.
